# Fast, Non-Competitive and Reversible Inhibition of NMDA-Activated Currents by 2-BFI Confers Neuroprotection

**DOI:** 10.1371/journal.pone.0064894

**Published:** 2013-05-31

**Authors:** Zhao Han, Jin-Long Yang, Susan X. Jiang, Sheng-Tao Hou, Rong-Yuan Zheng

**Affiliations:** 1 Department of Neurology, The First Affiliated Hospital and Research Institute of Experimental Neurobiology, Wenzhou Medical College, Wenzhou, Zhejiang Province, P. R. China; 2 Institute for Biological Sciences, National Research Council of Canada, Ottawa, Ontario, Canada; Federal University of Rio de Janeiro, Brazil

## Abstract

Excessive activation of the N-methyl-D-aspartic acid (NMDA) type glutamate receptors (NMDARs) causes excitotoxicity, a process important in stroke-induced neuronal death. Drugs that inhibit NMDA receptor-mediated [Ca^2+^]i influx are potential leads for development to treat excitotoxicity-induced brain damage. Our previous studies showed that 2-(2-benzofu-ranyl)-2-imidazoline (2-BFI), an immidazoline receptor ligand, dose-dependently protects rodent brains from cerebral ischemia injury. However, the molecular mechanisms remain unclear. In this study, we found that 2-BFI transiently and reversibly inhibits NMDA, but not AMPA currents, in a dose-dependent manner in cultured rat cortical neurons. The mechanism of 2-BFI inhibition of NMDAR is through a noncompetitive fashion with a faster on (K_on_ = 2.19±0.33×10^−9^ M^−1^ sec^−1^) and off rate (K_off_ = 0.67±0.02 sec^−1^) than those of memantine, a gold standard for therapeutic inhibition NMDAR-induced excitotoxicity. 2-BFI also transiently and reversibly blocked NMDA receptor-mediated calcium entry to cultured neurons and provided long-term neuroprotection against NMDA toxicity *in vitro*. Collectively, these studies demonstrated a potential mechanism of 2-BFI-mediated neuroprotection and indicated that 2-BFI is an excellent candidate for repositioning as a drug for stroke treatment.

## Introduction

Excitotoxicity takes place primarily as the result of glutamate binding to NMDAR and, to a lesser extent, by binding to other receptor subtypes. Over-stimulation of the NMDAR with L-glutamate or NMDA results in an excessive influx of Ca^2+^. Increased intracellular Ca^2+^ levels activate a plethora of potentially neurotoxic mechanisms, such as the early induction of a calcium-dependent protease, calpain, which cleaves intracellular structural proteins such as spectrin, causing the collapse of intracellular structures and eventually neuronal death [1]–[5]. Excitotoxicity contributes to a wide range of neurological disorders, such as stroke. Pharmacological inhibition of NMDAR ameliorates excitotoxicity-mediated neuronal death and protects the brain after cerebral ischemia [5].

However, since glutamate is the major excitatory transmitter in the brain, generalized inhibition of a glutamate receptor subtype like the NMDAR causes side effects that clearly limit the potential for clinical applications. Both NMDA and glycine competitive antagonists, even though effective in preventing glutamate-mediated neurotoxicity, cause a generalized inhibition of NMDAR activities and thus have failed in many clinical trials. It is believed that chemical molecules with a relatively fast off rate, and which only transiently blocks NMDA receptors, promise to be potential drug candidates for excitotoxicity-evoked brain damage. Drugs with these types of properties are expected to have the least side effects to the normal brain cognitive functions [6]–[11]. A prototype of such an uncompetitive, fast off-rate NMDA blocker is memantine which blocks NMDARs preferentially when they are excessively open. Memantine blocks high, pathological levels of agonist more effectively than lower, physiological levels [6], [7], [12]–[14]. Importantly, memantine potently, voltage- and concentration-dependently blocks human NMDA (GluN1/GluN2A) receptors [12]–[14]. As such, memantine is approved for treatment of Alzheimer’s disease and is currently undergoing numerous clinical trials including vascular dementia and stroke.

Our recent studies showed that ligands to the type 2 immidazoline receptor (I_2_R), such as 2-(2-benzofu-ranyl)-2-imidazoline (2-BFI) and Idazoxan, are potently and dose-dependently neuroprotective against transient cerebral ischemia, a rodent model of stroke [15]–[17]. These compounds readily cross the blood brain barrier as they were used as probes for IR studies [18]. I_2_R ligands directly bind to NMDA receptors [19] and may block NMDA receptor-gated calcium channels [20]–[23] in a manner similar to that of memantine [23]. Amongst I_2_R ligands, 2-BFI was the most effective in neuroprotection against glutamate toxicity *in vitro* [23] and cerebral ischemia *in vivo* [15]–[17]. However, the molecular mechanisms of 2-BFI-mediated neuroprotection against excitotoxicity remain unclear.

In this study, we used electrophysiological and cellular biology techniques to demonstrate that 2-BFI is a fast, non-competitive and reversible inhibitor of NMDARs. These evidence provided a potential mechanistic insight to explain neuroprotection conferred by 2-BFI in stroke brain.

## Materials and Methods

### Drugs and Chemicals

All chemicals and reagents, unless stated otherwise, were purchased from Sigma Chemical Co. (Saint Louis, MO). 2-BFI hydrochloride was purchased from Tocris Bioscience (Bristol, UK) and dissolved in water and was further diluted in the external solution to the final concentrations used in the experiments. All medium and supplements used for cell culture were purchased from Invitrogen (Shanghai, China). Cell permeable Fura-2-acetoxymethyl ester (Fura-2 AM) was purchased from Molecular Probes (Invitrogen Canada, Mississauga, ON, Canada).

### Experimental Animals

All animal procedures were conducted following an institutionally approved protocol in accordance with guidelines set by the National Institutes of Health Guidelines for the Care and Use of Laboratory Animals. All efforts were made to minimize suffering and to reduce the number of animals used. Healthy male Sprague-Dawley rats (3 to 4 months old at 250–280 g body weight) were obtained from the Experimental Animal Center of Wenzhou Medical College. All animals were fed with food and water *ad librum* and housed under a 12 h per day light-dark cycle.

### Rat Cortical Neuronal Culture

Primary cortical neurons were prepared from embryonic E18 Sprague-Dawley rats and cultured in neurobasal medium supplemented with B27 [24]. Briefly, cortices were explanted and cleaned free of meninges. The cortices were placed in D-Hanks’ solution and digested at 37°C with 0.05% trypsin-EDTA for 6 min. They were subsequently resuspended in DMEM medium supplemented with 20% fetal calf serum and 1% penicillin/streptomycin to stop digestion and were further dissociated into individual cells by trituration and plated on poly-D-lysine-coated glass coverslips in culture dishes at a density of 7×10^5^ cells/ml. After the neurons had attached to the coverslips for 2 hrs, the medium was changed to neurobasal medium containing 2% B27 supplement. Neurons were incubated at 37°C in a humidified atmosphere of 5% CO_2_ for 7–8 days before electrophysiological experiments.

### Whole-cell Electrophysiological Recordings

Whole-cell patch-clamp recordings were carried out at room temperature (22–25°C) using an Axopatch 700A patch-clamp amplifier (Axon Instruments, Inverurie, Scotland). Data acquisition was achieved using a DigiData 1322A with pClamp 9.0 software. The acquisition rate was 10 kHz and signals were filtered at 5 kHz. Patch electrodes were pulled with a Flaming/Brown micropipette puller (Sutter Instruments, Novato, CA) and fire-polished. The recording electrodes had a resistance of 4–6 MΩ when filled with different internal solutions. For the voltage-clamp recordings, the capacity transients were cancelled using the resistance capacitance circuit within the amplifier. After the formation of whole-cell configuration, access resistances were generally <15 MΩ. Series resistance compensation was set to 70%–90%. The liquid junction potential was approximately 2 mV and was auto-adjusted each time by pipette offset. To record NMDA/AMPA-activated currents, the external solution [(containing (mM): NaCl 150, KCl 5, CaCl_2_ 0.2, glucose 10 and HEPES 10, pH adjusted to 7.4 with NaOH)] and the pipette solution [containing (mM): KCl 140, MgCl_2_ 2.5, HEPES 10, EGTA 11, ATP 5, pH adjusted to 7.3 with KOH] were used. For voltage-clamp recordings, the membrane potential was held at −70 mV, unless noted otherwise. Drug solutions were prepared in extracellular solutions and applied to neurons by pressure using the 8-Channel Focal Perfusion System (ALA Scientific Instruments, Farmingdale, NY). Neurons were bathed constantly in extracellular solution between drug applications. Drug solution exchange was accomplished by electronic control.

Patch-clamp data was processed using Clampfit 9.0 (Axon Instruments) and then analyzed in Origin 7.5 (OriginLab, Northampton, MA). The dose-response curve was fitted to the logistic equation: *y* = (*A_1_*–*A_2_*)/[1+ (*x*/*x_0_* )*^p^*]+*A_2_*, where *y* is the response, *A_1_* and *A_2_* are the maximum and minimum response, respectively, *x_0_* is the concentration corresponding to half-maximal effect, *x* is the drug concentration, and *p* is the Hill coefficient.

The onset and offset rates of 2-BFI were measured from the recordings by the binding kinetic protocol, where a single concentration of 2-BFI was applied in the constant presence of agonists. Tau_on_ and Tau_off_ were obtained by a single exponential function fit: *I* = *A*×[1-exp(-*t*/τ)]+*I*_0_, where *I* is the current, *I*_0_ is the steady-state amplitude of the current, *A* is the difference between the peak and steady state current amplitudes, *t* is time, and τ is the time constant.

### Neuronal Viability Assay

After 7 days-in-vitro, cortical neurons were treated with the specific inhibitor for 15 min prior to the addition of 100 µM glutamate or 200 µM NMDA at 37°C. The plates were then incubated for up to 24 h at 37°C in the presence or absence of inhibitors. Untreated cells were also included as controls. At the end of the treatment period, cells were either fixed for staining or subjected to a neuronal viability assay using Alamar Blue (Invitrogen). Stained cells were examined under a fluorescent microscope (Carl Zeiss, AX10 vert 200M), and digital images were taken and analyzed using Image J software (http://rsbweb.nih.gov/ij/). The viability of cortical neurons treated with NMDA, and with or without inhibitors as mentioned, was assayed using an Alamar Blue assay (Invitrogen). Briefly, a 1∶10 dilution of Alamar blue was added to cells for 1 h at 37°C. One third of the medium was removed and read in a 96-well plate using a plate reader with λ_Ex_ = 530 nm and λ_Em_ = 590 nm. At minimum, a triplicate reading was obtained per experiment with three independent repeats.

### Ratiometric Measurement of [Ca^2+^]i using Fura-2

Ratiometric measurement of [Ca^2+^]i was performed using Fura-2 AM [25]. Briefly, mouse cortical neurons at 7 days-in-vitro on glass coverslips were loaded with 5 µM Fura-2-AM (Molecular Probes, Eugene, CA) plus 0.02% pluronic (Life Technologies, INC, Burlington, ON, Canada) for 30 min at 37°C. After rinsing with PSS Mg^2+^ free buffer containing 2 mM HEPES (pH 7.2), 140 mM NaCl, 5 mM KCl, 2.3 mM CaCl_2_, and 10 mM glucose, and stabilized in the same buffer for 5 min, Fura-2 intensities were measured using a Northern Eclipse Digital Ratio Image System (EMPIX, Mississauga, ON, Canada) with an Axiovert 200 camera and light source (Zeiss, Thornwood, NY). Fura-2 fluorescence was measured at 510 nm emission with 340/380 nm dual excitation selected by a DG-5 system (Sutter Instrument Company, Novato, CA). [Ca^2+^]i concentration was represented by the ratio of florescence intensity between the two excitation wavelengths of R340/380 of Fura-2 after correction for background. R340/380 for 10 cells in one field of each coverslip was averaged. The basal level of [Ca^2+^]i was recorded for 20 sec, followed by the application of inhibitors. NMDA (200 µM) was dissolved in PSS buffer and was added to cortical neurons with or without inhibitors. [Ca^2+^]i was recorded for 60–100 sec. After washing with PSS buffer for 300 sec, PSS buffer containing 45 mM KCl was added to neurons to record changes in [Ca^2+^]i for 60 sec to show viability of neurons. All measurements were repeated for at least 3 times. The data was analyzed using Microsoft Excel and presented as the mean of three experiments.

### Western Blotting

Western blotting procedures were as previously described [26]. Protein samples (10 µg) from control and treated neuronal cultures, prepared in lysis buffer (10 mM HEPES, pH 7.9; 10 mM KCl; 1.5 mM MgCl_2_; 0.1 mM EDTA; 10% glycerol; 0.1% NP40; 0.3% Triton X-100, and proteases inhibitor cocktails), were boiled in loading buffer (62.5 mM Tris, pH 6.8; 10% glycerol, 2% SDS, 0.01% bromophenol blue and 5% β-mercaptoethanol) prior to being loaded onto a 10% SDS-polyacrylamide gel for electrophoresis and Western blotting. Proteins were electro-blotted onto a nitrocellulose membrane in ice-cold transfer buffer (192 mM glycine, 25 mM Tris buffer with 20% methanol) for 1 h. Membranes were blocked with 5% skimmed milk in Tris-buffered saline with Tween-20, containing 10 mM Tris.HCl, pH 7.6; 150 mM NaCl; 0.1% Tween-20, for at least 30 min before an overnight incubation at 4°C with selected primary antibodies diluted (1∶100 to 1∶1000) in the blocking buffer. Next day, the membranes were rinsed for 30 min in Tris-buffered saline with Tween-20 and bands were visualized with HRP-conjugated secondary antibody (raised against rabbit, mouse or goat, depending upon the species in which the primary antibody was raised). Enhanced chemiluminescence detection of the target proteins was achieved using the ECL detection system from GE Healthcare Life Sciences (Mississauga, ON, Canada).

### Data Analysis and Statistics

Results were expressed as means ± SEM. Statistical analysis of the differences between groups was carried out using a Student’s *t*-test (paired *t*-test) or one-way ANOVA followed by Tukey’s *post hoc* test. *P* values of <0.05 were considered statistically significant.

## Results

### Inhibition of NMDA-activated Currents by 2-BFI

**Figure 1:**
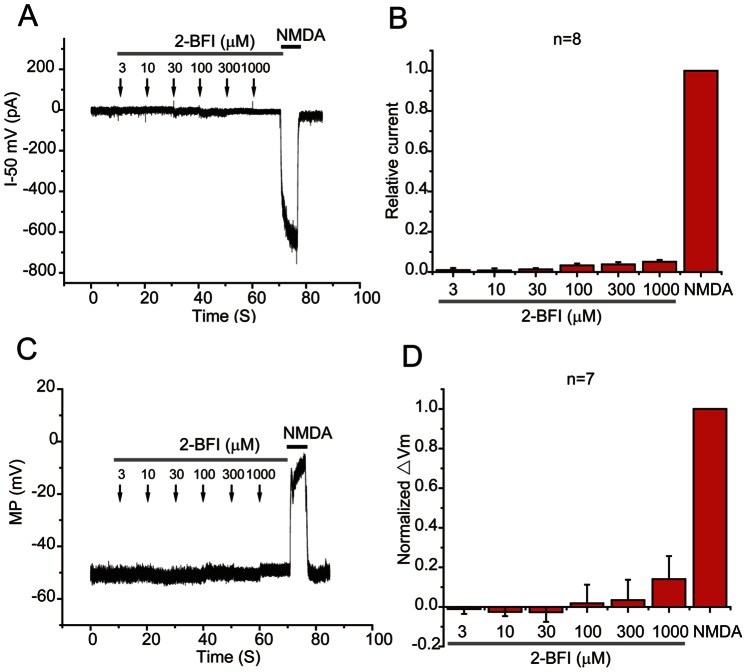
Effects of 2-BFI and NMDA on rat cortical neuronal excitability. (A) Representative current traces induced by 3–1000 µM 2-BFI and 30 µM NMDA plus 1 µM glycine at −50 mV are shown. (B) The current amplitude is normalized to the current activated by NMDA and the summary data is shown. (C) Representative membrane potential (MP) change induced by 3–1000 µM 2-BFI and 30 µM NMDA plus 1 µM glycine is shown. (D) The membrane potential change (ΔVm) is normalized to that of NMDA and the summary data is shown. Data in B and D represents the mean ± SEM.

The effect of 2-BFI and NMDA on neuronal excitability was first determined in cultured cortical neurons. As shown in Fig. 1A and 1C, 30 µM NMDA plus 1 µM glycine induced a large inward current at −50 mV and depolarized the membrane from −50 mV to −10 mV. In contrast, application of 2-BFI with a series of concentrations in the range of 3–1000 µM neither induced any detectable current at −50 mV, nor affected the membrane potential. The average current amplitude was normalized to the current activated by NMDA as shown in Fig. 1B and D, which indicated that 2-BFI does not affect cortical neuronal membrane potential.

**Figure 2:**
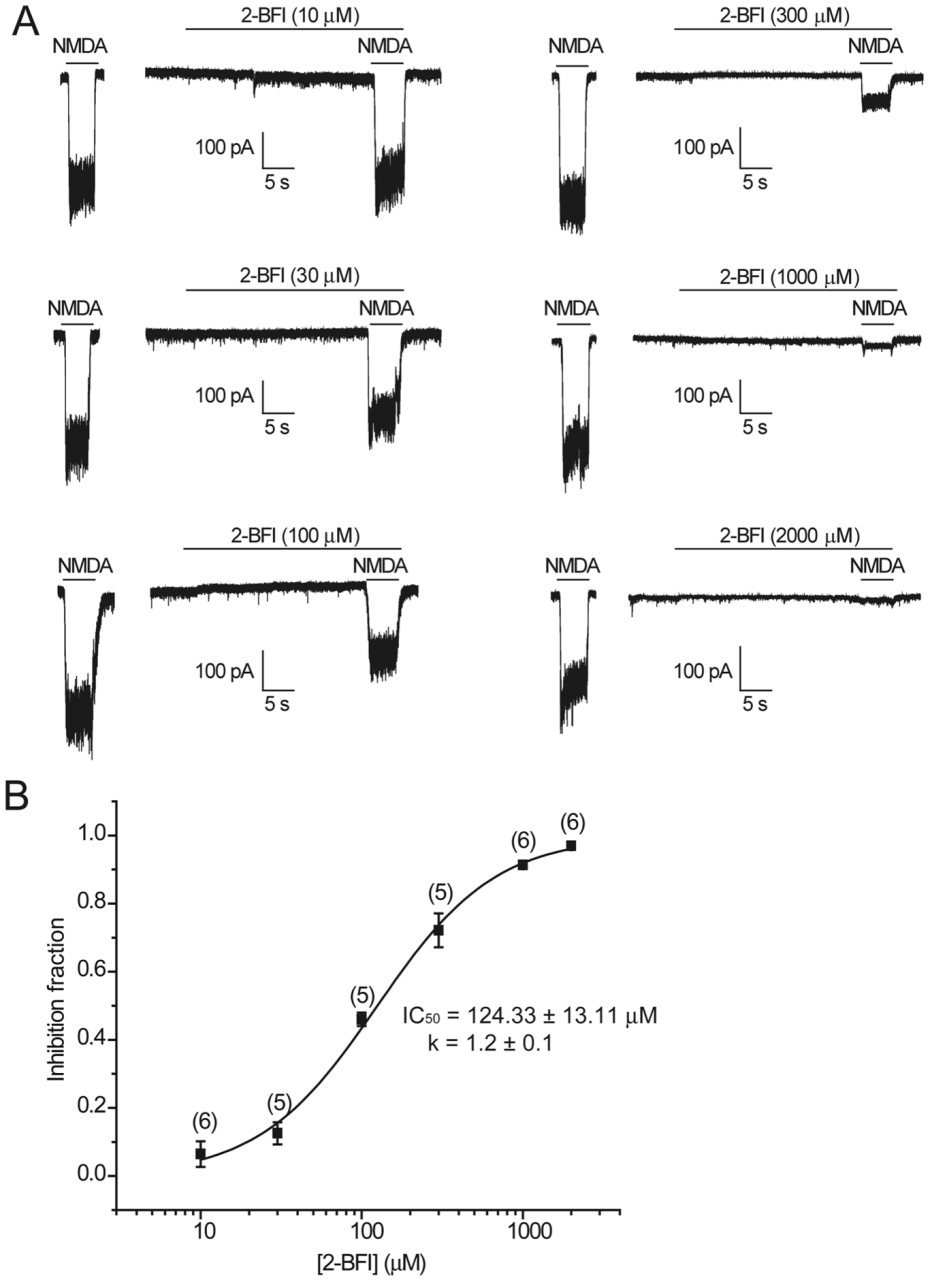
Inhibition of NMDA-activated current by 2-BFI in rat cortical neurons. (A) Representative currents activated by 30 µM NMDA plus 1 µM glycine and their inhibition by 10–2000 µM 2-BFI. 2-BFI was first applied for 30 s, and then simultaneously applied with NMDA. Membrane potential was clamped at −70 mV. (B) 2-BFI concentration-response relationship for inhibition of NMDA currents activated by 30 µM NMDA plus 1 µM glycine. Concentration-response curve was fitted by the logistic equation, with the IC_50_ of 124.33±13.11 µM and a slope factor of 1.2±0.1. Data presented in B represents the average of 5–6 cells ± SEM.

To investigate the effect of 2-BFI on NMDA-activated current, the dose-response relationship was established by pre-application of 2-BFI for 30 s before simultaneously applying with NMDA (Fig. 2A). 2-BFI inhibited NMDA-activated current in a dose-dependent manner (Fig. 2B) over the range of 10–2000 µM with an IC_50_ of 124.33±13.11 µM and the slope factor of 1.2±0.1 (n = 6). This inhibition was fully reversible after a few seconds of washing. These experiments showed that 2-BFI not only dose-dependently inhibited NMDA current, but also in a reversible manner, as when 2-BFI and NMDA were removed, the membrane potential returned fully to the normal resting level.

**Figure 3:**
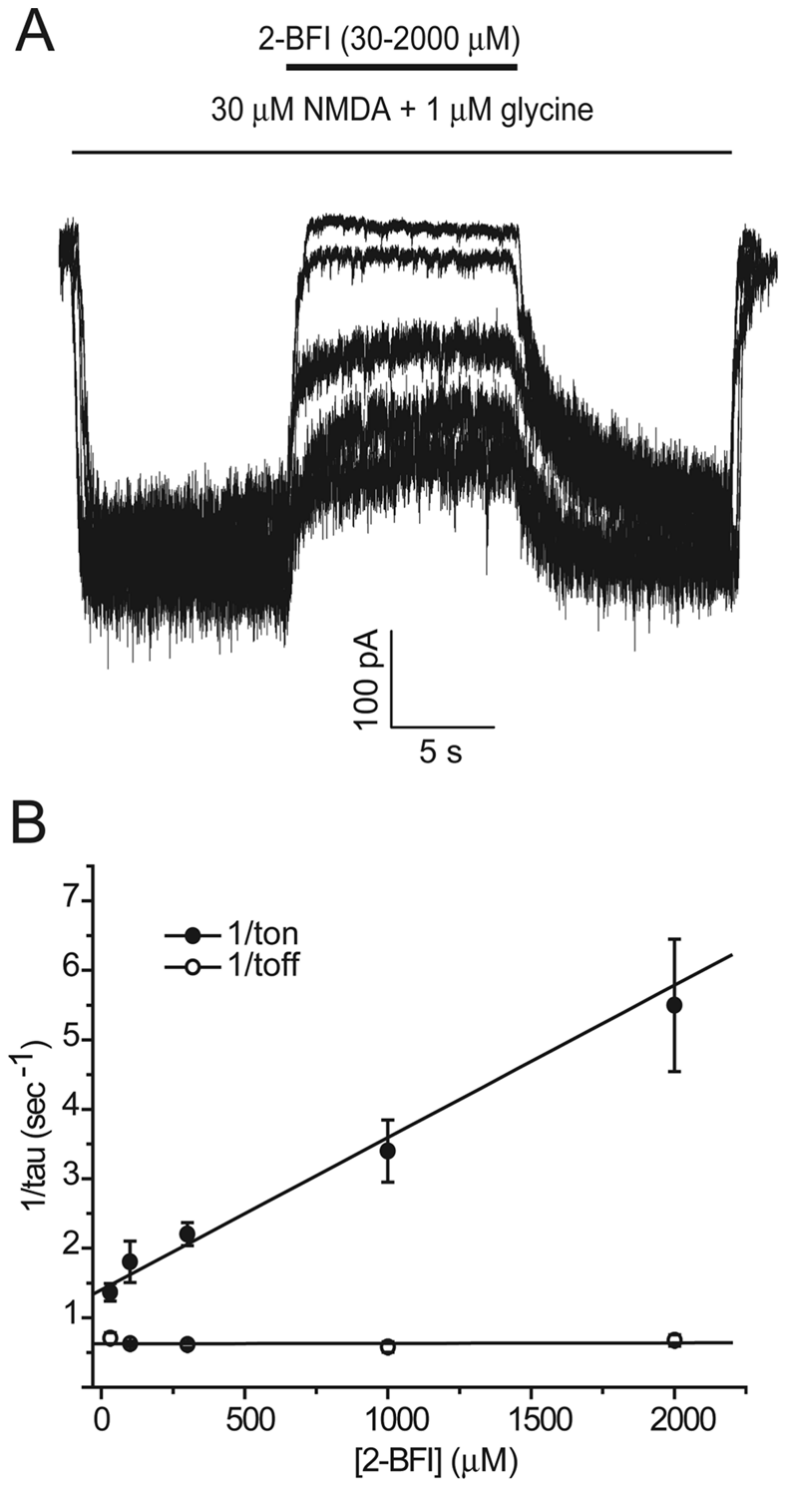
The binding kinetics of 2-BFI on NMDA receptors. (A) Representative current traces showing the kinetic protocol, where a single concentration of 2-BFI was applied in the constant presence of 30 µM NMDA plus 1 µM glycine. (B) The onset and offset rates of 2-BFI were measured from the recordings by the kinetic protocol. Tau_on_ and Tau_off_ were obtained by a single exponential function fit. Mean 1/tau values were plotted against the corresponding 2-BFI concentration to determine K_on_ and K_off_. The fitted kinetic values were K_on_ = 2.19±0.33×10^−9^ M^−1^sec^−1^, K_off_ = 0.67±0.02 sec^−1^, Kd = K_off/_K_on_ = 305.94±0.3 µM.

To demonstrate whether 2-BFI reversibly blocks NMDAR and to evaluate the affinity of 2-BFI for NMDA receptors, the binding kinetics were determined using the protocol shown in Fig. 3A. NMDA current was stimulated using 30 µM NMDA and 1 µM glycine followed by the addition of 2-BFI. In the kinetic experiments, 2-BFI showed a single exponential blocking kinetics. The fitted kinetic values were K_on_ = 2.19±0.33×10^−9^ M^−1 ^sec^−1^, K_off_ = 0.67±0.02 sec^−1^, Kd = K_off/_K_on_ = 305.94±0.3 µM. The Kd value was consistent with the measured IC_50_ value (Fig. 3B), albeit that the Kd value was slightly higher than IC_50_ value, suggesting the existence of potential spare receptors. Nevertheless, more importantly, these data showed that 2-BFI behaved as a reversible NMDAR blocker at all the doses with a fast on-and-off rate.

### Voltage-independent Inhibition of NMDA-activated Currents by 2-BFI

**Figure 4:**
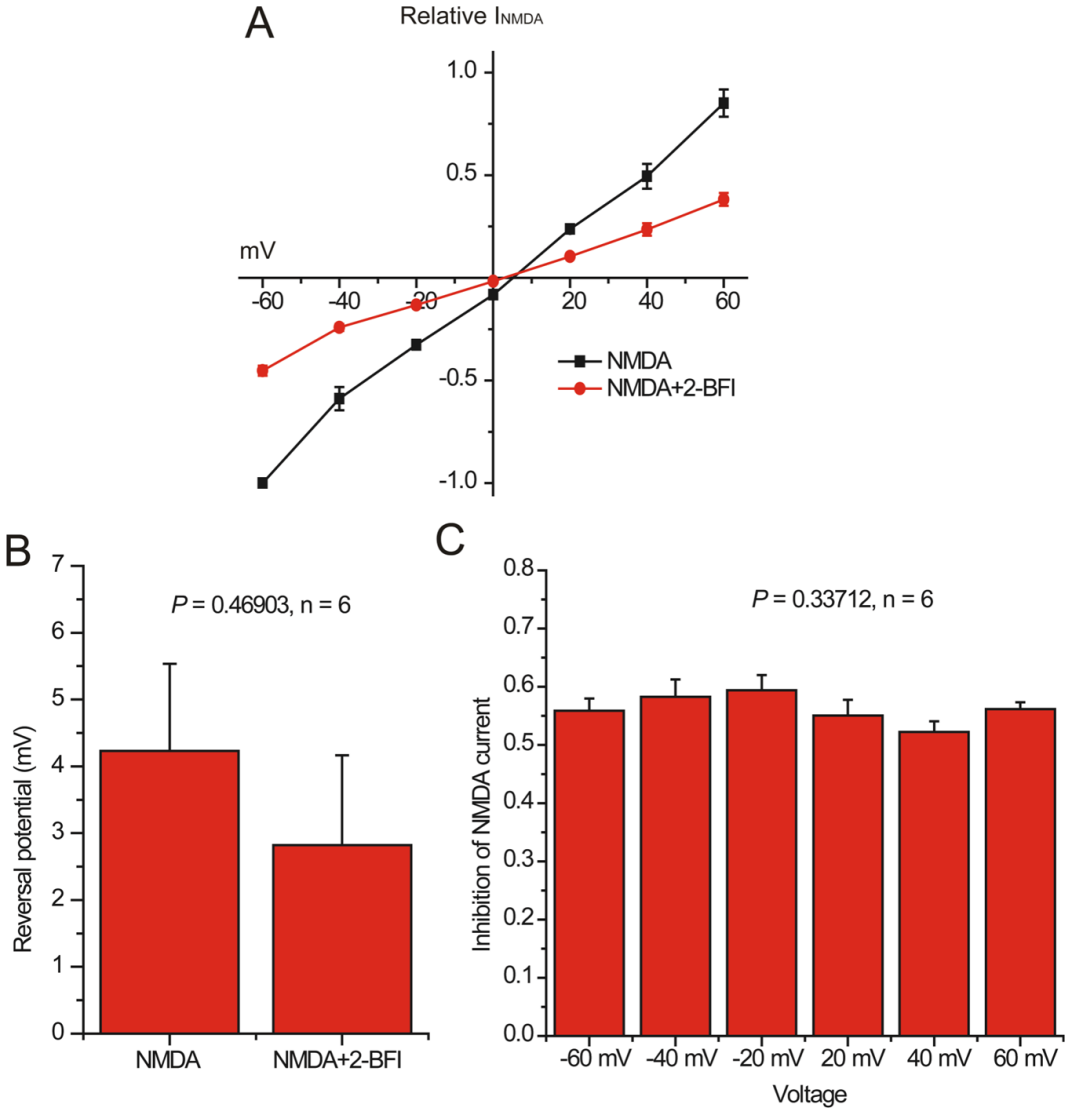
Effects of membrane potential on inhibition of NMDA-activated current by 2-BFI in rat cortical neurons. (A) Current-voltage relationship for currents activated by 30 µM NMDA and 1 µM glycine in the absence or the presence of 200 µM 2-BFI at membrane potentials between −60 mV and +60 mV. Current amplitude is normalized to the current activated by 30 µM NMDA and 1 µM glycine in the absence of 2-BFI at −60 mV. (B) The average reversal potential of current activated by 30 µM NMDA and 1 µM glycine was 4.2±3.2 mV in the absence and 2.8±3.2 mV in the presence of 200 µM 2-BFI (paired t-test with *P*>0.05, n = 6). (C) Summary data showing inhibition of NMDA-activated current by 200 µM 2-BFI at membrane potentials from −60 mV to +60 mV. The percentage inhibition of NMDA-activated current by 2-BFI was not significantly different as determined using one way ANOVA with *post hoc* Tukey’s analysis (*P*>0.05, n = 6).

To examine whether inhibition of NMDA-activated channels by 2-BFI was voltage-dependent, the amplitude of NMDA-activated peak current was measured over a range of holding potentials in the absence or the presence of 2-BFI. The current-voltage relationship as shown in Fig. 4A demonstrated that the NMDA-activated currents in the presence of 2-BFI showed a similar voltage-dependency as that of the NMDA only control, but without altering the reversal potential (Fig. 4B, Student’s *t*-test, *P*>0.05, n = 6). Summary data, shown in Fig. 4C, also demonstrated that the average inhibition of NMDA-activated current by 200 µM of 2-BFI at membrane potentials from −60 mV to +60 mV was not significantly different (ANOVA, *P*>0.05, n = 6), demonstrating that 2-BFI inhibition of NMDA current was voltage independent.

### Inhibition of NMDA-activated Currents by 2-BFI was Non-competitive with Agonists

**Figure 5:**
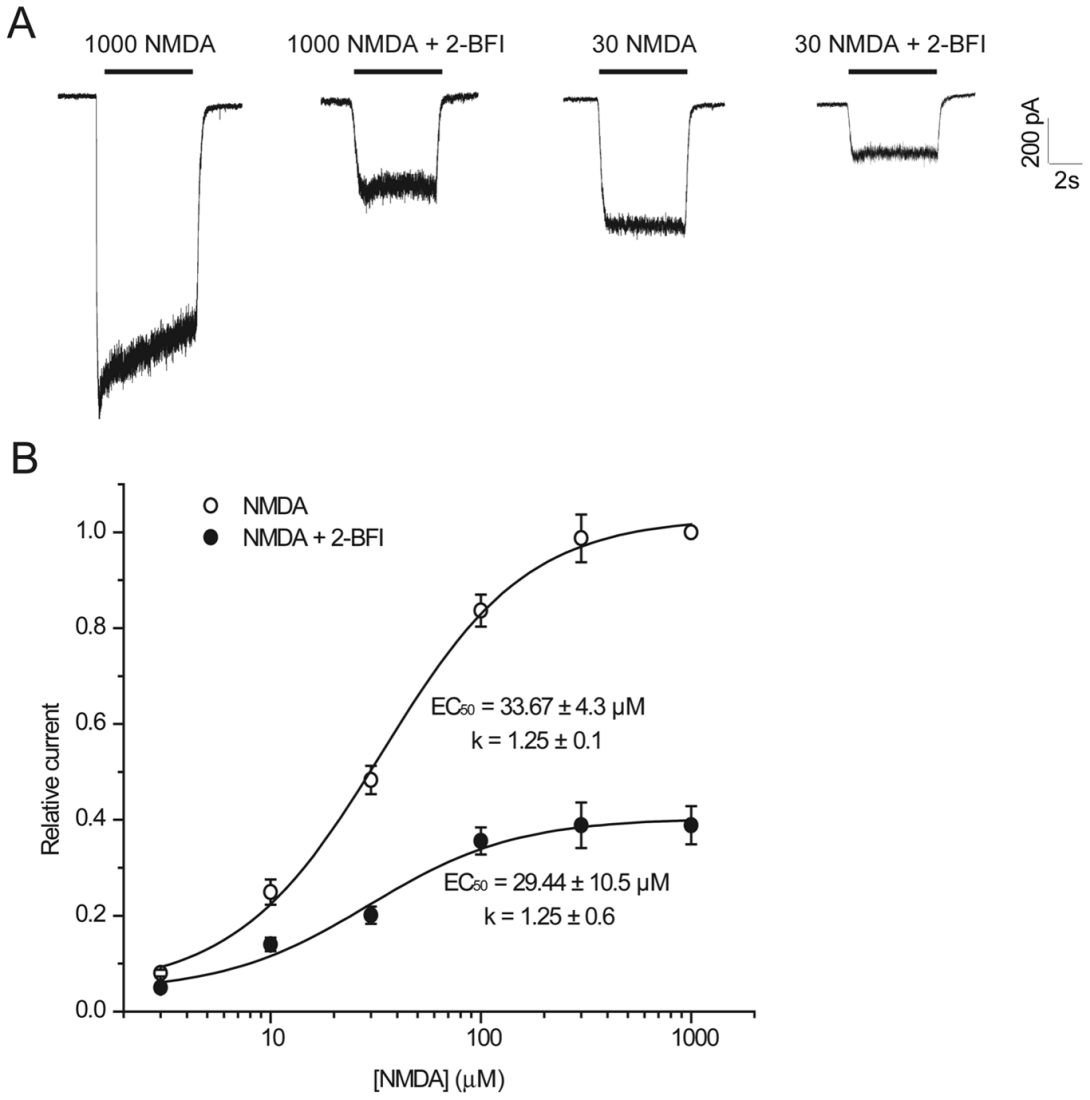
Effect of NMDA concentration on 2-BFI inhibition of NMDA-activated current in rat cortical neurons. (A) Representative currents activated by 1000 µM NMDA and 30 µM NMDA in the presence of 1 µM glycine before and after application of 200 µM 2-BFI in a single cell. 2-BFI was first applied for 30 s, and then simultaneously applied with NMDA. (B) Concentration-response relationship for NMDA in the absence (○) and presence (•) of 200 µM 2-BFI. Current amplitude is normalized to the current activated by 1000 µM NMDA and 1 µM glycine. Concentration-response curves were fitted by logistic equation, with the EC_50_ of 33.67±4.3 µM and the slope factor of 1.25±0.1 in the absence of 2-BFI and EC_50_ of 29.44±10.5 µM and the slope factor of 1.25±0.6 in the presence of 2-BFI (n = 6–8).

**Figure 6:**
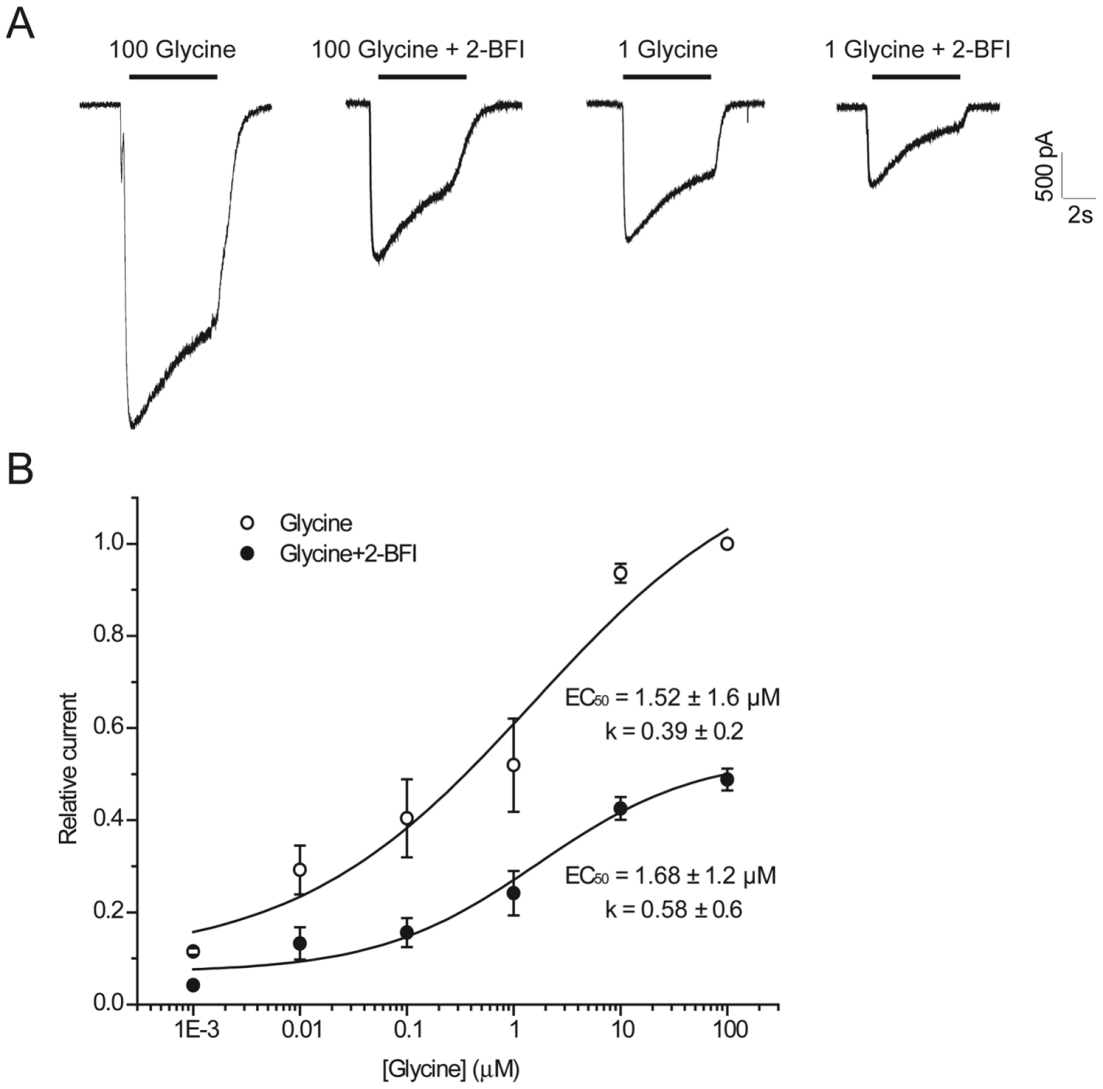
Effect of glycine concentration on 2-BFI inhibition of NMDA-activated current in rat cortical neurons. (A) Representative currents activated by 100 µM glycine and 1 µM glycine in the presence of 30 µM NMDA before and after the application of 200 µM 2-BFI in a single cell. 2-BFI was first pre-applied for 30 s, and then simultaneously applied with glycine. (B) Concentration-response relationship for glycine in the absence (○) and presence (•) of 200 µM 2-BFI. Current amplitude is normalized to the current activated by 100 µM glycine and 30 µM NMDA. Concentration-response curves were fitted by the logistic equation, with the EC_50_ of 1.52±1.6 µM and the slope factor of 0.39±0.2 in the absence of 2-BFI and EC_50_ of 1.68±1.2 µM and the slope factor of 0.58±0.6 in the presence of 2-BFI (n = 6∼8).

To determine whether 2-BFI inhibition of NMDA current is competitive or non-competitive, we examined whether the concentration of NMDA or glycine affects 2-BFI inhibition by determining the EC_50_s of NMDA or glycine action in the absence or the presence of 200 µM 2-BFI. As shown in Fig. 5A, the 2-BFI inhibition was not reduced by increasing the concentration of NMDA from 30 µM to 1000 µM. The dose-response relationship (Fig. 5B) revealed that the EC_50_s of NMDA (33.67±4.3 µM vs. 29.44±10.5 µM) or the slope factor of the curve (1.25±0.1 vs. 1.25±0.6) were not different in the absence or the presence of 200 µM 2-BFI. Glycine exhibited a similar effect to that induced by NMDA. As shown in Fig. 6A, the inhibition by 200 µM 2-BFI was not reduced by increasing the concentration of glycine from 1 µM to 100 µM. The dose-response relationship (Fig. 6B) revealed that the EC_50_s of glycine (1.52±1.6 µM vs. 1.68±1.2 µM) or the slope factor of the curve (0.39±0.2 vs. 0.58±0.6) did not differ in the absence or the presence of 200 µM 2-BFI. The findings that 2-BFI greatly reduced the Emax of NMDA and glycine without altering the EC_50_s or the slope factors suggested that 2-BFI acted as a non-competitive antagonist of NMDAR and did not influence the binding of glycine.

### Selectivity of 2-BFI for iGluRs and Ion Channels in Cortical Neurons

**Figure 7:**
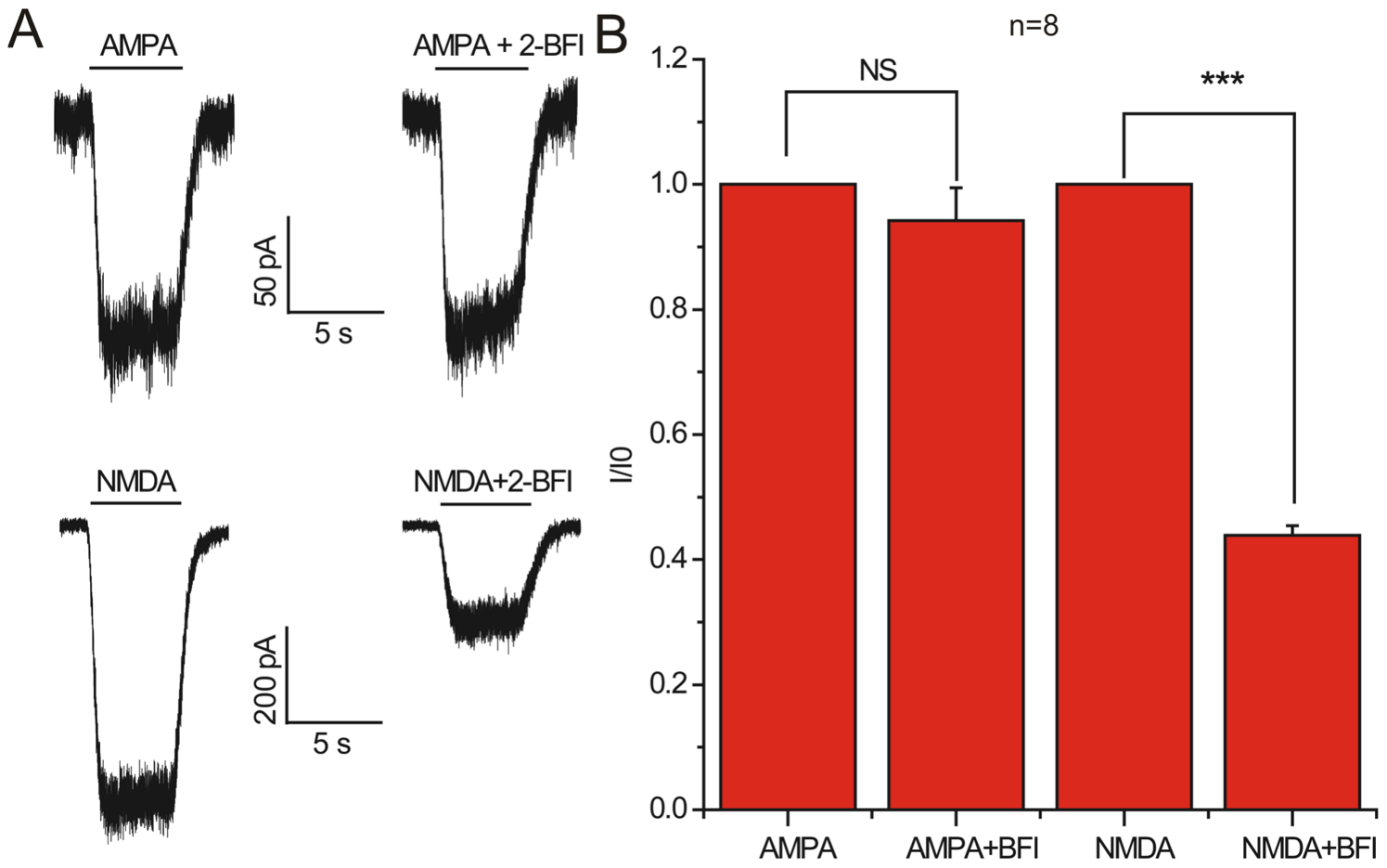
Effect of 2-BFI on AMPA-activated current in rat cortical neurons. (A) Representative currents activated by 100 µM AMPA and 30 µM NMDA plus 1 µM glycine and their inhibition by 200 µM 2-BFI. (B) Summary data for inhibition of AMPA-activated current and NMDA-activated current by 2-BFI. 2-BFI at 200 µM significantly reduced NMDA-activated current, but had no effect on AMPA-activated current.

The strong inhibition of NMDA-induced currents by 2-BFI led us to ask whether 2-BFI affected other ionotropic glutamate receptors, such as AMPA. As shown in Fig. 7, in contrast to the marked inhibition of NMDA-induced currents by 2-BFI (paired *t*-test, *P*<0.001, n = 8), 2-BFI did not inhibit AMPA-induced currents (Fig. 7B, paired *t*-test, *P*>0.05, n = 8), indicating the relative selectivity of 2-BFI against NMDAR compared to AMPA receptor.

### 2-BFI Attenuates NMDA-induced [Ca^2+^]i Response in Cortical Neurons in a Transient and Reversible Manner

**Figure 8:**
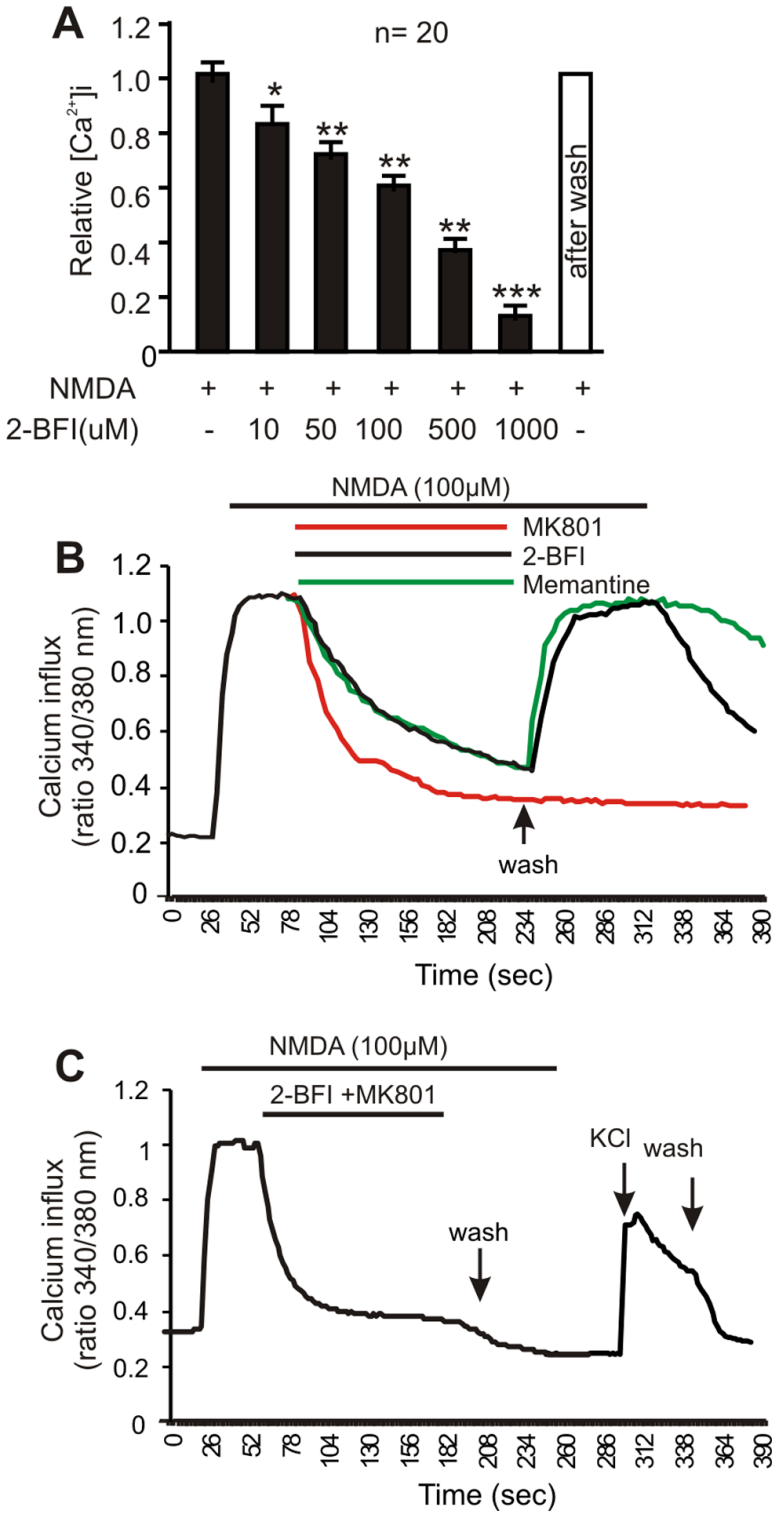
Inhibition of NMDA-evoked [Ca^2+^]i in cortical neurons. (A) Relative [Ca^2+^]i level in the presence of various doses of 2-BFI and 100 µM NMDA was normalized to NMDA evoked [Ca^2+^]i. Data represents the average of measurements of at least 20 cells. (B) Representative traces of [Ca^2+^]i in cortical neurons treated with MK801 (10 µM), 2-BFI (200 µM) or memantine (10 µM) in the presence of NMDA (100 µM). A brief wash with PSS removed the inhibition of NMDA-induced [Ca^2+^]i in 2-BFI and memantine treated cells, but not in MK801 treated cells. (C) Adding 2-BFI and MK801 together completely inhibited NMDA induced [Ca^2+^]i and such an inhibition can not be removed by washing. Adding KCl depolarized the membrane and showed a non-regulated calcium influx.

To demonstrate whether 2-BFI inhibits NMDA-evoked calcium response, cortical neurons were treated with 100 µM NMDA in the presence of a range of concentrations of 2-BFI (10–1000 µM). As shown in Fig. 8A, 2-BFI significantly suppressed [Ca^2+^]i influx at concentrations between 10–1000 µM (n = 20 cells per dose point; ANNOVA with *post hoc* Tukey’s analysis, * indicates *P*<0.05, ** indicates *P<*0.01, while *** indicates *P*<0.001). Most interestingly, after washing, the [Ca^2+^]i influx in cortical neurons was restored to the level comparable to that of NMDA only treatment (Fig. 8A), suggesting transient and reversible inhibition by 2-BFI.

This transient and reversible inhibition property of 2-BFI was compared with other known non-competitive NMDAR inhibitors MK801 and memantine (Fig. 8B). In contrast to MK801, which non-reversibly blocked NMDA receptor-mediated [Ca^2+^]i influx, cortical neurons treated with 2-BFI and memantine were able to respond to NMDA (100 µM) almost immediately when 2-BFI and memantine were removed from the cell culturing buffer (Fig. 8B; n = 20 cells per treatment). When 2-BFI and MK801 were applied together to cortical neurons, washing will not be able to remove non-reversible blocking of NMDAR by MK-801 (Fig. 8C). These studies demonstrated that 2-BFI has similar characteristics to memantine in transient and reversible blocking NMDAR-mediated [Ca^2+^]i influx in cortical neurons.

### 2-BFI Protection of Cultured Cortical Neurons against NMDA Excitotoxicity

**Figure 9:**
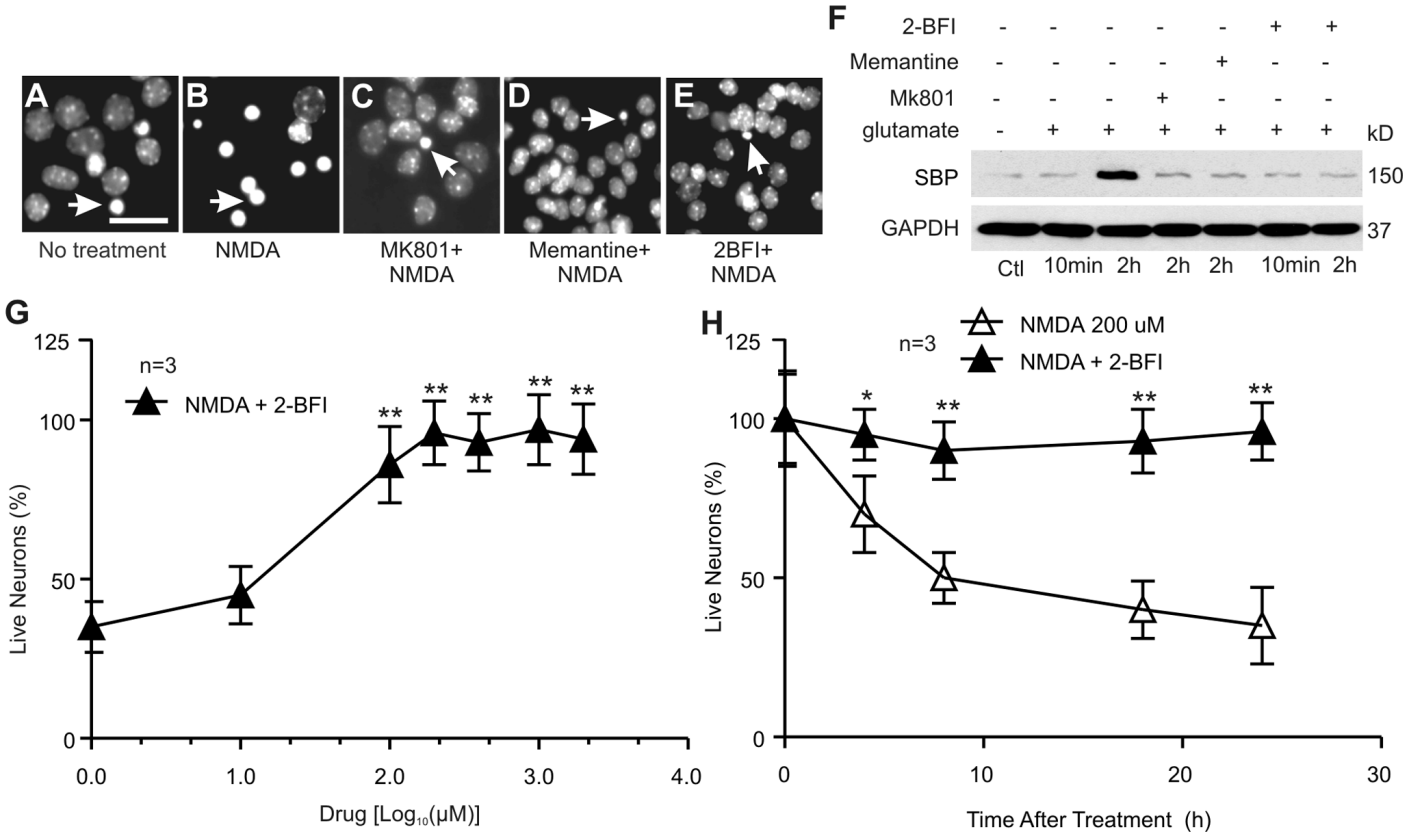
Neuroprotection by 2-BFI against NMDA toxicity. (A–E) Photomicrographs of nuclei of cortical neurons treated with or without NMDA and other indicated inhibitiors. Arrows show condensed nuclei, indicating dead cells. Scale bar = 100 µm. (F) Treated cortical neurons were subjected to Western blotting to show inhibition of the production of spectrin breakdown product (SBP) induced by NMDA excitotoxicity. GAPDH was used as an internal protein loading control. (G) Dose-dependent neuroprotection against NMDA by 2-BFI was measured using an Alamar blue assay the mean ± SEM was plotted (n = 3). (H) Time-dependent protection by 2-BFI against NMDA toxicity to cortical neurons was measured using an Alamar blue assay. The data represents mean ± SEM of at least three repeats. ** indicates statistical significant using paired t-test (*P*<0.01).

The protective effect by 2-BFI against NMDA-induced excitotoxicity in cortical neurons was examined using nuclear staining (Figs. 9A-E) and an Alamar blue assay (Figs. 9 G and H). NMDA-induced neuronal death showed typical condensed nuclei (arrows in Fig. 9B-E) and the number of which were reduced when treated with MK801, memantine and 2-BFI, indicating neuroprotection. Neuronal death after 2 h treatment with NMDA also produced elevated spectrin breakdown product (SBP), serving as a marker for increased neuronal death (Fig. 9F). 2-BFI, memantine and MK801 effectively reduced the level of SBP, again confirming neuroprotection. The Alamar blue assay also showed clear dose- and time-dependent protection against NMDA toxicity (paired *t*-test, ** indicates *P*<0.01, n = 8).

## Discussion

In the present study, we demonstrated that 2-BFI, an I_2_R ligand, is a fast, non-competitive and reversible inhibitor of NMDARs during excitotoxicity. Electrophysiological data showed that 2-BFI is a negative allosteric modulator of NMDAR which is noncompetitive to NMDA and glycine. Ratiometric calcium measurement further confirmed the function of 2-BFI in reducing NMDAR-mediated influx of toxic level of calcium into neurons. These studies therefore provided a potential molecular mechanism for 2-BFI neuroprotection against glutamate toxicity *in vitro* (Fig 9) and cerebral ischemia *in vivo* as we have previously demonstrated [15]–[17].

The inability of 2-BFI to affect the membrane potential of neurons (Fig. 1), fast kinetics and reversibility of NMDA current inhibition (Fig. 3), voltage independence of the inhibitory action (Fig. 4), independence on agonist concentration (Fig. 5), and receptor selectivity at least in respect to AMPAR (Fig. 7), all supported the idea that 2-BFI is a fast non-competitive reversible inhibitor of NMDARs. Together with the fact that 2-BFI is able to cross the blood brain barrier [18], our data suggested that 2-BFI an excellent candidate for further development as a drug for stroke therapeutics. 2-BFI is a fast and reversible NMDAR blocker with superior binding kinetics to NMDAR compared to that of memantine. 2-BFI not only inhibits NMDA currents when applied prior to NMDA, but is also effective when applied after the opening of the NMDA channel. The fitted kinetic values for 2-BFI interaction with the NMDAR are K_on_ = 2.19±0.33×10^−9^ M^−1^ sec^−1^, K_off_ = 0.67±0.02 sec^−1^, while in contrast, the K_on_ for memantine at 12 µM is 1 s and the K_off_ is about 5 s. A relatively fast off-rate is a major contributor to a drug’s low affinity for the channel pore. It has been proposed that a clinically tolerated neuroprotective drug would consist of a low-affinity, open-channel blocker with a relatively fast off-rate [9], [10], [12]–[14], [27]. Such a drug would not substantially interfere with normal synaptic neurotransmission by accumulating in the channels. As a result, the drug would be both effective and well tolerated. In comparison with memantine, we can speculate that 2-BFI would be equally well tolerated clinically. In fact, our previous animal studies showed that 2-BFI administration has no visible side effects to rodents [15]–[17], [28], [29]. Indeed, 2-BFI is tolerated by neurons and animals even at high doses. 2-BFI at doses ranging from 3–1000 µM (Figs. 1–2), showed no obvious interference with the resting membrane potential, yet, dose-dependently inhibited NMDA currents in the presence of NMDA (Figs 8 and 9). In comparison with memantine, the Ic_50_ of 2-BFI at −70 mV is much higher than that of memantine (2-BFI is 124.33±13.11 µM v.s. 1.2 µM for memantine). However, this relatively high concentration of 2-BFI appeared to have no adverse effect on neurons.

There are three types of imidazoline receptors based on imidazoline binding sites: I_1_ site is labeled by clonidine and the I_2_ site is labeled by idazoxan and other selective molecules such as 2-BFI. A putative I_3_ site has also been described [30]. So far only ligands to I_2_R, such as 2-BFI and idazoxan, have been found to prevent ischemia-hypoxia induced neuronal damage both *in vitro* and *in vivo* [16], [19], [20], [22], [23]. Because 2-BFI has been used to characterize and assess the nature of I_2_-imidazoline receptors in rat brain and liver [18], it is believed that 2-BFI can readily cross the blood brain barrier. In fact in the cerebral cortex, 2-BFI displayed high affinity (Ki = 9.8 nM) for a single class of [3H]2-BFI binding sites [18].

The mode and site of interaction between 2-BFI and NMDAR, however, remain unclear. Whether 2-BFI neuroprotection was exclusively due to the inhibition of NMDAR after cerebral ischemia is also not unclear. In particular, the measured Kd value at 305.94±0.3, which is higher than the IC_50_, suggests the existence of spare receptors in the system. Other calcium-permeable channels and routes of calcium entry, such as transient receptor potential channels and acid-sensing ion channels are also known to contribute to the excitotoxicity [31]–[33]. Whether 2-BFI affects these channels remains to be investigated.

Stroke is the leading cause of death and disability in the developed world and is rapidly becoming the No. 1 killer in developing countries such as China [5], [34], [35]. However, stroke therapeutics still represent the largest unmet medical need. So far, apart from thrombolytic drugs, there are no effective treatments for stroke-induced brain damage in spite of tremendous progress made in the understanding of the fundamental mechanism of neuronal death caused by stroke [5], [34]. Chemical compounds directly blocking glutamate NMDA receptors have so far failed to show efficacy in human stroke clinical trials due to side effects resulting from interference with the normal physiological functions of the NMDA receptors [36], [37]. Drugs which uncompetitively or noncompetitively inhibit NMDAR are good candidates for stroke treatment [6], [12]–[14]. 2-BFI with its characteristics of fast, reversible, and non-completive binding to NMDAR, and effective brain protection against stroke [15]–[17] promises to be a clinically tolerated effective drug for stroke treatment.
